# COGNITIVE INTRAINDIVIDUAL VARIABILITY IN ADULTS WITH AND WITHOUT HIV-ASSOCIATED NEUROCOGNITIVE DISORDER

**DOI:** 10.1093/geroni/igae098.2781

**Published:** 2024-12-31

**Authors:** David Vance, Jason Blake, Wei Li, Pariya Wheeler, Christopher Collette, Alexandra Jacob, Vidyulata Kamath, Victor Del Bene

**Affiliations:** University of Alabama at Birmingham, Birmingham, Alabama, United States; University of Alabama at Birmingham, Birmingham, Alabama, United States; University of Alabama at Birmingham, Birmingham, Alabama, United States; University of Alabama at Birmingham, Birmingham, Alabama, United States; Emory University, Atlanta, Georgia, United States; University of Alabama at Birmingham, Birmingham, Alabama, United States; Johns Hopkins University School of Medicine, Baltimore, Maryland, United States; University of Alabama at Birmingham, Birmingham, Alabama, United States

## Abstract

Cognitive intra-individual variability (IIV) is a sensitive marker of reduced top-down cognitive control related to neuropathology and increases with age. Prior work has shown increased cognitive IIV in people living with HIV (PLWH). For those with HIV-Associated Neurocognitive Disorder (HAND), it is likely that cognitive IIV is greater compared to PLWH without HAND (Non-HAND). In a sample of older PLWH from the Deep South of the United States, we examined differences in cognitive IIV in those with and without HAND, and the relationship of IIV with clinical and demographic factors. HAND (n=108) and Non-HAND (n=23) participants completed a neuropsychological assessment as part of a larger cognitive training protocol. Our primary outcome measure was the coefficient of variation (CoV), a widely used measure of IIV that corrects for mean-performance. Group differences were examined with analysis of covariance, followed by correlations of CoV with clinical and demographic variables. Cognitive IIV was greater in the HAND than the Non-HAND Group, consistent with our hypothesis. When exploring demographic variables included as covariates, we found race was statistically significant. Specifically, Black individuals with HAND had greater cognitive IIV than White individuals with HAND. However, these observed race differences in cognitive IIV were no longer significant after including reading ability and years of education in the model. Relative to PLWH without HAND, those with a HAND diagnosis have greater cognitive IIV attributable to more advanced HIV and cerebral dysfunction. Race-related differences in cognitive variability are best explained by historical disparities in education and reading levels.

